# Identification and Application of BhAPRR2 Controlling Peel Colour in Wax Gourd (*Benincasa hispida*)

**DOI:** 10.3389/fpls.2021.716772

**Published:** 2021-10-01

**Authors:** Lianlian Ma, Zhengguo Liu, Zhikui Cheng, Jiquan Gou, Jieying Chen, Wenjin Yu, Peng Wang

**Affiliations:** ^1^College of Agriculture, Guangxi University, Nanning, China; ^2^Institute of Vegetable Research, Guangxi Academy of Agricultural Sciences, Nanning, China

**Keywords:** *APRR2*, chlorophyll, frameshift mutation, peel color, wax gourd

## Abstract

Peel color is an important factor affecting commodity quality in vegetables; however, the genes controlling this trait remain unclear in wax gourd. Here, we used two F_2_ genetic segregation populations to explore the inheritance patterns and to clone the genes associated with green and white skin in wax gourd. The F_2_ and BC_1_ trait segregation ratios were 3:1 and 1:1, respectively, and the trait was controlled by nuclear genes. Bulked segregant analysis of both F_2_ plants revealed peaks on Chr5 exceeding the confidence interval. Additionally, 6,244 F_2_ plants were used to compress the candidate interval into a region of 179 Kb; one candidate gene, *Bch05G003950* (*BhAPRR2*), encoding two-component response regulator-like protein *Arabidopsis* pseudo-response regulator2 (APRR2), which is involved in the regulation of peel color, was present in this interval. Two bases (GA) present in the coding sequence of *BhAPRR2* in green-skinned wax gourd were absent from white-skinned wax gourd. The latter contained a frameshift mutation, a premature stop codon, and lacked 335 residues required for the protein functional region. The chlorophyll content and *BhAPRR2* expression were significantly higher in green-skinned than in white-skinned wax gourd. Thus, *BhAPRR2* may regulate the peel color of wax gourd. This study provides a theoretical foundation for further studies of the mechanism of gene regulation for the fruit peel color of wax gourd.

## Introduction

Wax gourd [*Benincasa hispida* (Thunb.; Cogn.; Cucurbitaceae)] is an annual herb that has been cultivated in China for ~1,500years and has an annual planting area of >200,000 hm^2^. Peel color is an important commodity quality used to judge fruit maturity and thus influences consumer preferences. The peel color of Chinese wax gourd may vary: dark or light green, cyan-green, or white. Variability in the chlorophyll, carotenoid, and anthocyanin contents accounts for the color differences among cultivars (Li et al., 2007; Zhu et al., 2016). Although white-skinned wax gourd is rare, its superior appearance and dense mouthfeel are favored by consumers. Therefore, peel color genetics and fine mapping and cloning of the genes regulating this trait have important theoretical and practical implications for accelerating the molecular breeding of wax gourd cultivars that have a high commodity value.

Several studies have investigated pericarp color inheritance in Cucurbitaceae. Cucumber pericarp may be dark or light green, white, or orange. Yellow-green (*yg*) cucumber pericarp is recessive to dark green and is epistatic to light green (Pierce and Wehner, 1990; Xie and Wehner, 2001; Dong et al., 2012). *Csa7G05143* and *Csa6G133820* are associated with the light green pericarp trait (Lun et al., 2015; Zhou et al., 2015). Li et al. (2013) studied the inbred cucumber lines WI7200 (blackthorn and orange peel color) and WI7201 (whitethorn and cream peel color) and found that the former had a single base insertion in the third intron of *R2R3-MYB* that modulates peel color by regulating anthocyanin biosynthesis. However, the white peel trait in immature cucumber was regulated by *w* in the *Arabidopsis* pseudo-response Regulator2 (*APRR2*) family. A single G insertion in this gene in white cucumber caused early translation termination, which was primarily responsible for the white peel trait (Liu et al., 2016). Green, yellow, and white melon peel colors have been extensively studied. Oren et al. (2019) identified two variant alleles in *CMAPR2* (*MELO3C003375*) in dark and light green melon. A C→G mutation in exon 8 or 13bp insertion in exon 9 terminated *CMAPR2* translation. Both mutations may account for the light green skin color in melon. Ou (2019) found that a 13bp deletion in *CMAPR2* explained non-green melon skin colors. *CmKFB,* which encodes the F-box protein, is a post-transcriptional negative regulator of naringenin chalcone accumulation and is associated with yellow melon peel formation. A 12bp insertion in the 5′-untranslated region of *CmKFB* downregulated the gene and promoted flavonoid accumulation in yellow-peeled melon (Feder et al., 2015). Zhao et al. (2019) performed a genome-wide association study of the genes regulating peel color in 635 melon accessions and demonstrated that *CMAPR2* (*MELO3C003375*) on Chr4 and *CMKFB* (*MELO3C011980*) on Chr10 are the major regulators of melon peel color.

Dark and light green and yellow watermelon peel colors have been extensively investigated. In a study of light and dark green watermelon skin, Oren et al. (2019) found that *CLAPRR2* controls peel color. An A G→C mutation in the *CLAPRR2* intron prematurely terminates variant transcript translation in light green watermelon. Through fine mapping, Li et al. (2019) identified *CLCG08G01780* as a major candidate gene regulating watermelon peel color. The coding sequence (CDS) of *CLCG08G01780* harbors a C→G nonsynonymous mutation in light green peel and encodes a 2-phytate-1,4-β-naphthoquinone methyltransferase protein that participates in chlorophyll and photosystem I biosynthesis. Studies have also focused on the peel color of related species. For example, pumpkin peel color regulation is complex. Over 20 loci control this trait, including B (bicolor yellow; (Shifriss, 1955, 1981), Y (yellow; Scarchuk, 1954; Paris et al., 2004), W (weak fruit coloration; (Shifriss, 1955), D (dark green stem; Shifriss, 1981; Paris, 2003), L-1 (light fruit color-1), and L-2 (light pigmentation on fruit-2; (Globerson, 1969; López-Anido et al., 2003).

Fruit peels vary in color because of the relative differences in their chlorophyll, carotene, anthocyanin, and flavonoid contents. Several pigmentation-regulating genes have been identified, including *MYB* transcription factors (TFs), *KNOX*, and *APRR2* TFs. The plant *MYB* TF family is large and regulates secondary pathways of phenylpropanoid metabolism and flavonoid and anthocyanin biosynthesis. These TFs are important for pulp, peel, and leaf coloration (Takos et al., 2006; Ban et al., 2007; Espley et al., 2007; Azuma et al., 2008; Chen et al., 2009). The *GLK2* TF belongs to the GARP superfamily of MYB class TFs (Riechmann et al., 2000) and has been reported in maize (Langdale and Kidner, 1994), *Arabidopsis* (Fitter et al., 2002), *Capsicum annuum* L (Brand et al., 2014), and *Lycopersicon esculentum* (Powell et al., 2012; Nguyen et al., 2014). *GLK2* is a conserved master TF that regulates chloroplast development in fleshy fruit (Jia et al., 2020). *KNOX* genes may play active roles in the influence of phytohormonal cytokine on chloroplast biogenesis, development, and activity. *KNOX* genes may control fruit but not leaf chloroplast development (Richmond and Lang, 1957; Mothes, 1960; Kusaba et al., 1998; Ori et al., 1999; Giovanna et al., 2001; Tanaka et al., 2011; Nadakuduti et al., 2014; Cortleven and Schmülling, 2015). A study of tomato showed that *TKN2* and *TKN4* were expressed in a latitudinal gradient during fruit ripening and caused a gradient in chloroplast development. Another study of tomato revealed that *TKN2* promotes fruit chloroplast development by regulating *SLGLK2* and *SLAPRR2* expression (Nadakuduti et al., 2014). *APRR2* may be a member of the pseudo-response regulator (PRR) subfamily of response regulators (RRs; Satbhai et al., 2011). These two-component regulatory systems in the signal transduction machinery mediate output signals (Makino et al., 2000). Pan et al. (2013) studied tomato and proposed that *APRR2* regulates plastid development and maturation. Liu et al. (2016) suggested that *APRR2* positively regulates phytohormonal cytokines. *APRR2* shares high homology with *GLK2;* however, only the former may play a role in maturation (Pan et al., 2013). *GLK2* expression was unchanged in plants with upregulated *APRR2*, and *APRR2*-like expression was unchanged in plants overexpressing *GLK2* (Carvalho et al., 2011; Powell et al., 2012; Pan et al., 2013). Hence, *APRR2* and *GLK2* are mutually independent TFs that induce genes controlling chloroplast development in fruit (Nadakuduti et al., 2014). Nevertheless, the regulatory mechanism of *APRR2* remains unclear.

Several preliminary studies of the genetics of wax gourd peel color showed that dark green is dominant over lighter or yellow-green and that this trait is controlled by a pair of nuclear genes (Li et al., 2007; Deng et al., 2015). The wax gourd peel color gene (dark green vs. yellow) was first mapped to Chr5 by (Jiang et al., 2015). However, genetic research using molecular markers has not been extensively conducted to explore wax gourd coloration. In the present study, we used two crosses between green- and white-skinned wax gourd cultivars to identify the key genes responsible for wax gourd peel color. We combined phenotyping, genotyping, gene mapping, and candidate gene analysis. The aim of this study was to provide a theoretical basis for the functional validation of the genes regulating wax gourd fruit rind color and to guide molecular marker-assisted breeding of high-quality wax gourd cultivars.

In recent years, due to the development in biotechnology, a novel genotyping technology kompetitive allele-specific PCR (KASP) marker, based on single nucleotide polymorphisms (SNPs), used to successfully map single genes has been reported in many crops, such as in wheat (Youfu et al., 2021), rice (MeSun et al., 2021), melon (Yanyan et al., 2021), and maize (Babadev et al., 2020). Cleaved amplified polymorphic sequences (CAPS) markers have been widely used in molecular marker-assisted breeding. This technique has had profound applications in tomato (Ashish et al., 2021), rapeseed (Matuszczak et al., 2020), and maize (Pan et al., 2021). In this study, we conducted a genotype-phenotype joint analysis by KASP markers, compressing the mapping interval. CAPS markers were then developed for application in molecular marker-assisted breeding of wax gourd.

## Materials and Methods

### Plant Material

**Figure 1:**
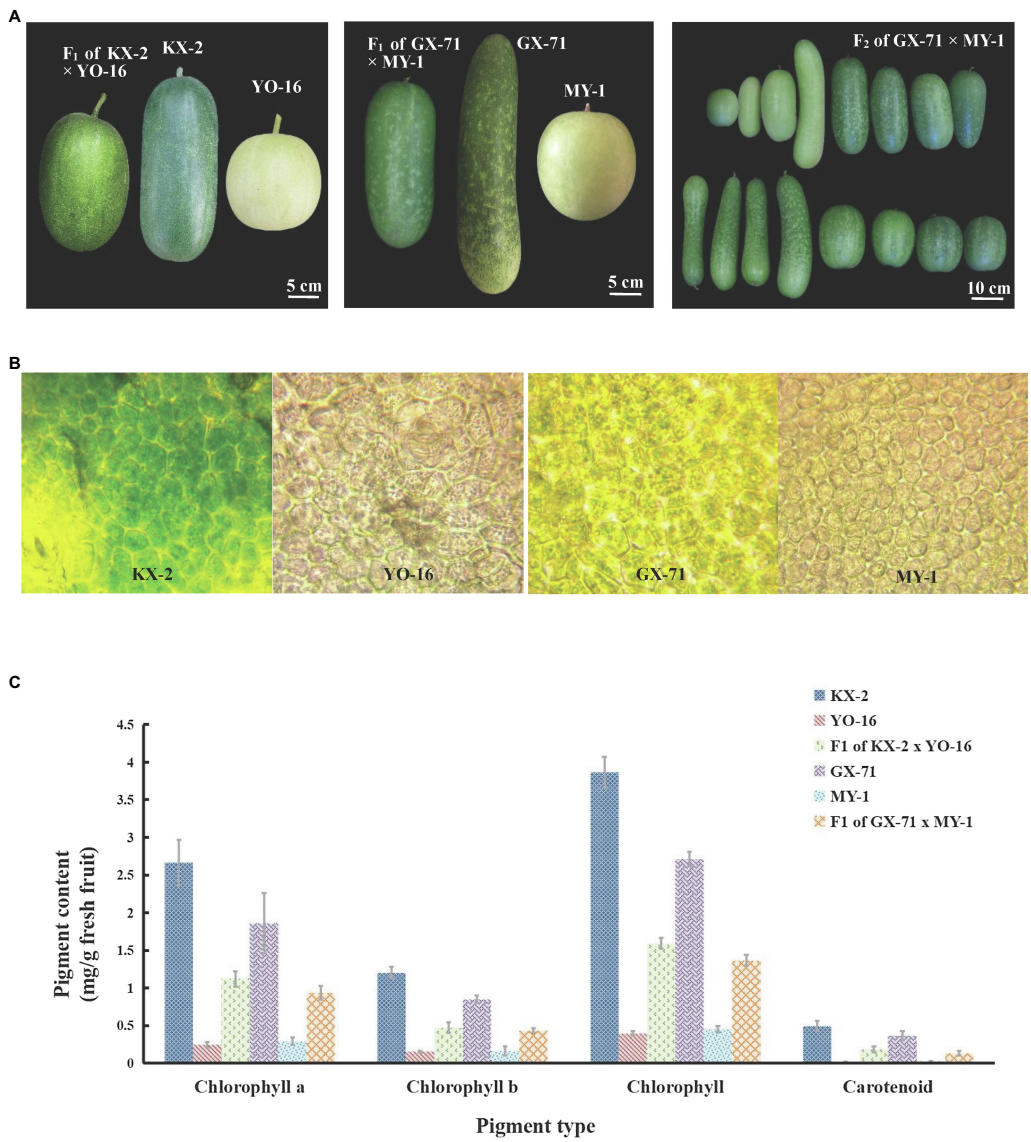
Pericarp phenotypic characteristics and chloroplast number and pigment content of wax gourd. **(A)** Phenotypic characteristics of two hybridization KX-2×YO-16 and GX-71×MY-1 and their F_1_ plant and F_2_ of GX-71×MY-1. **(B)** Chloroplast observation of four parents, from left to right are KX-2,YO-16,GX-71,MY-1. **(C)** Pigment content of four parents (KX-2×YO-16 and GX-71×MY-1) and their F1.

The parental wax gourd lines ink green KX-2 (male) and green-skinned GX-71(male) and the white-skinned lines YO-16(female) and MY-1(female) were used (Figure 1A). KX-2 was long and cylindrical with an inky green peel, no spines, and marginated seeds. GX-71 was long and rod-shaped with a dark green peel, spotted, and spineless. Both white-skinned parents were round in shape, white, and spineless. All four lines were provided by Kenong Seed Co., Ltd (Nanning, Guangxi, China).

The hybridizations KX-2×YO-16 and GX-71×MY-1 generated F_1_ plants which were then self-pollinated to generate F_2_ plants. F_1_ plants of both crosses were backcrossed to white-skinned recessive parents to obtain BC_1_ populations. The parental, F_1_, F_2_, and BC_1_ populations were used to describe and validate wax gourd green/white color inheritance. F_2_ plants were used to detect recombination events and narrow the mapping interval. Forty inbred lines were obtained from Kenong Seed Co., Ltd. and used to validate cleaved amplified polymorphic sequence markers. All plants were grown under natural sunlight at the experimental wax gourd bases at Shajing, Nanning, and Guangxi in China (108°51′ east longitude, 22°48′ north latitude).

### Phenotypic Data Collection and Analyses

Parental, F_1_, F_2_, and BC_1_ plants from two cross combinations had identification tags showing their serial numbers and pollination dates. The wax gourds used in phenotypic measurements had been pollinated for 15days to ensure stable peel color and chlorophyll content. After the fruits were harvested, statistical field phenotype data were collected. The color of the peel was observed using the naked eye. Color consistent with the white skin parent was recorded as white, and color consistent with the green skin parent was recorded as green. A chi-square goodness-of-fit test was used to analyze the segregation rates of the F_2_ and BC_1_ populations. Two or three young leaves were cut from the top of the parental and F_2_ plants, wrapped in aluminum foil, frozen in liquid nitrogen, and stored at-80°C for subsequent DNA extraction. The peels of the parents were cut with a sterile blade, quick-frozen in liquid nitrogen, and stored at −80°C until needed. Each plant retained only three wax gourds. Surplus fruits were removed to ensure the normal growth and development of the remaining fruit.

### Pigment Content and Chloroplast Observation

To observe the chloroplasts of epidermal cells, the tissue of four parents’ pericarp at 15days after pollination was sliced with a sterile blade, placed on a glass slide with a small drop of distilled water, pressed onto a coverslip, made into microscopic sections, observed under a light microscope (DM4000, Leica, Wetzlar, Germany) at 400×, and photographed.

The pericarp chlorophyll content was measured as described by Wilson et al. (2000). Three phenotypically normal wax gourds were harvested per plant. The surface of the pericarps was cleaned, and portions with normal color and no obvious color difference were selected. The pericarps were scraped off with a blade, and 0.3g tissue was weighed and placed in each 50-mL centrifuge tube. Next, 45ml of 95% (v/v) ethanol was quickly added and each tube was sealed with ParaFilm^™^ (Bemis, Neenah, WI, United States) and labeled. The control contained 200μl of 95% (v/v) ethanol. The tubes were placed on a shaker at 200rpm for 13h and centrifuged at 3821×g for 3min at 25°C. Each supernatant was transferred to a 200μl centrifuge tube and measured in triplicate in a microplate reader (Infinite 2000, TECAN). All manipulations were performed under low light. The wavelengths were 665, 649, and 470nm for chlorophylls a and b and carotenoid, respectively. The pigment content was determined using the following equations:

$$\begin{array}{l} \mathrm{Pigment content}\;\left(\mathrm{mg}/g\right)=\\ \left(\begin{array}{l} \mathrm{pigment concentration}\;\left(\mathrm{mg}/L\right)\times \\ \mathrm{extracted liquid product}\;\left(L\right)\times \mathrm{dilution factor} \end{array}\right)/\mathrm{sample weight}\;\left(g\right). \end{array}$$

$$\mathrm{Ca}\;\left(\mathrm{mg}/L\right)=13.95\times A665-6.88\times A649.$$

$$\mathrm{Cb}\;\left(\mathrm{mg}/L\right)=24.96\times A649-7.32\times A665.$$

$$\mathrm{Cc}\;\left(\mathrm{mg}/L\right)=1,000\times A470-2.05\times \mathrm{Ca}-114.8\times \mathrm{Cb}.$$

$$\begin{array}{l} \mathrm{Total chlorophyll content}\;\left(\mathrm{mg}/g\right)=\\ \mathrm{chlorophyll}\;a\;\mathrm{content}+\mathrm{chlorophyll}\;b\;\mathrm{content}. \end{array}$$

where Ca is the chlorophyll a concentration, Cb is the chlorophyll b concentration, Cc is the carotenoid concentration, A665 is the chlorophyll a absorbance at 665nm, A649 is the chlorophyll b absorbance at 649nm, and A470 is the carotenoid absorbance at 470nm.

### DNA Extraction

Genomic DNA was extracted from young leaves using the CTAB method as described by Clark et al. (1997). DNA was quantified using an ultra-micro spectrophotometer (K5800, KAIAO, Beijing, China) and evaluated by electrophoresis in a 1.2% agarose gel.

### Bulked Segregant Analysis Sequencing Mapping Strategy

Sixty F_2_ plants with extreme traits (30 white and 30 green) were selected from 1,244 F_2_ generated by GX-71×MY-1 to construct two extreme admixture pools. Similarly, 60 individual plants (30 white and 30 green) with extreme phenotypes were selected from 244 F_2_ plants generated by KX-2×YO-16 hybridization. The young leaves were collected to extract DNA, and the qualified DNA samples were used to construct a gene library, which was quality-checked. The qualified library was sequenced on Illumina HiSeq™ PE150 (San Diego, CA, United States) at 5x depth. The original image data files obtained by high-throughput sequencing were converted into sequenced reads after base calling. To ensure the quality of information analysis, we filtered the raw reads to obtain clean reads, which were used for subsequent information analysis. The main steps of data filtering were as follows: (1) remove reads with adapters; (2) filter reads with an N content of more than 10%; and (3) remove reads with a mass value of less than 10 bases and more than 50%. The white pools, green pools, and their two parent pools were used for association analyses, with GX-19 used as the reference genome. Resequenced data were compared against the available wax gourd reference genome (GX-19) using BWA software to identify reliable mutation information through a filter pipeline. Two association analysis methods, the Euclidean distance (ED) association algorithm and Δ (SNP/indel-index) algorithm, were used.

### Marker Development

To narrow the mapping interval, based on the bulked segregant analysis sequencing (BSA-seq) data, as well as the distribution and density of the SNP physical locations, 14 KASP markers were designed for each 1–4Mb interval in the preliminary mapping candidate interval. Preparation of the mixture for analysis and PCR amplification was performed according to the manufacturer’s instructions (LGC Genomics, Shanghai, China). The PCR reaction system occupied a volume of 3μl, including 1.0μl of DNA (8–15ngμL-1), 1.5μl of 2×master mix, and 0.5μl of primer mix. PCR amplification was performed using landing PCR. The reaction conditions were as follows: heat treatment at 95°C for 15min; denaturation at 95°C for 20s, annealing and extension between 65 and 55°C for 60s, ten landing cycles (each cycle reduced by 1.0°C); and denaturation at 95°C for 20s, annealing and extension at 57°C for 1min, 26cycles; followed by preservation in dark conditions at 4°C. After amplification, fluorescence scanning and genotyping were performed. We preliminarily designed 14 KASP markers; 1,244F_2_ plants were used for phenotype-genotype association analysis. To further narrow the scope of mapping, an F_2_ population containing 5,000 individuals was constructed, and we used flanking markers to genotype 5,000 F_2_ plants to identify the recombinants. New KASP markers were developed in the flanking marker to detect the genotype of the recombinant plant, and the genotype-phenotype joint analysis was used to infer the most likely target gene region.

### Sequencing Analysis of Candidate Gene

Sequences and gene functions were retrieved from the Cucurbit Genomics Database^1^ and available wax gourd reference genome (GX-19). The full-length CDS sequence was used for gene cloning and candidate gene sequence analysis. The primer sequences were designed by Primer 5 software and shown in Supplementary Figure 1. RNA was extracted from pericarps of four parental using an Eastep^®^ Super Total RNA extraction kit according to the manufacturer’s instructions., and first-strand cDNA was synthesized using a 5× All-in-One Master Mix kit and an AccuRT Genomic DNA Removal kit (Diamed Life Sciences, Mississauga, Ontario, Canada). The 2× A8 FastHiFi PCR Master Mix (Aidlab, Beijing, China) was used for PCR amplification. The PCR product was detected by 1.2% agarose gel electrophoresis, and the target strip was recovered and purified with an Axyprep DNA Gel Extraction Kit (Axygen, Union City, CA, United States). A zero-background pTOPO-Blunt cloning kit (CV16) from Aidlab (Beijing, China) was used to construct an expression vector according to the manufacturer’s instructions: 1μl of pTOPO-Blunt vector, 1μl of 10× Enhancer, and 40–80ng PCR gel products were mixed, and sterile water was added to a total volume of 10μl. This sample was ligated at 30°C for 5min. The vector was transformed into Trans5α chemically compatible cells according to the manufacturer’s instructions (TransGen Biotech, Beijing, China). All fragments were sequenced by Shanghai Shengong Biotechnology Co., Ltd (Shanghai, China). DNA and amino acid sequences were aligned using DNAMAN v.9 software (Lynnon Biosoft, San Ramon, CA, United States).

### RNA Extraction and RT-qPCR Analysis of Candidate Gene

Real-time quantitative PCR (RT-qPCR) was used to quantify developmental and tissue-specific expression of candidate genes. RNA was extracted from four parents’ pericarps (0–15days after pollination) and other tissues (root, stem, leaf, male flower, female flower, and flesh) using an Eastep^®^ Super Total RNA extraction kit according to the manufacturer’s instructions. First-strand cDNA was synthesized with 5× PrimeScript^™^ RT Master Mix (Perfect Real Time; TaKaRa Bio, Shiga, Japan). RT-qPCR was performed with PrimeScript^™^ RT Master Mix (Perfect Real Time; TaKaRa Bio). The RT-qPCR was run in a volume of 10μl containing 1μl cDNA template, 0.4μl of each primer (10μm), 5μlTB Green Premix Ex TaqII (2×; Tli RNaseH Plus; TaKaRa Bio), 0.2μl ROX Reference Dye II (50×; Thermo Fisher Scientific, Waltham, MA, United States), and 6μl RNase-free water. The PCR protocol was as follows: 95°C for 30s; 40cycles of 95°C for 5s, 60°C for 34s; and 95°C for 15s, 60°C for 1min, and 95°C for 15s.

AP-2 complex subunit mu *(CAC, Bch05G003650)* was used as the normalization control for RT-qPCR across different samples. Primer sequences for the internal reference gene and *BhAPRR2* are shown in Supplementary Figure 1. The values of three reactions were averaged, and relative expression was determined using the 2^−ΔΔCt^ method (Livak and Schmittgen, 2001). The experiment was conducted using an Applied Biosystems 7,500 Real-Time PCR instrument (Foster City, CA, United States).

### Phylogenetic Analyses

The *APRR2* genes of some other Cucurbitaceae crops and *Arabidopsis thaliana* with high homology to the *BhAPRR2* gene were downloaded from NCBI^2^ in FASTA format. These sequences were compared with clustalW, and then, the phylogenetic tree was constructed with MEGA 6.0 with the neighbor-joining method (Li et al., 2015). Bootstrapping was performed with 1,000 replicates. The sequence accession numbers are shown in Supplementary Figure 8.

### Molecular Marker-Assisted Selection Test

A CAPS marker was developed according to the candidate gene sequence by Primer 5 software. The primer sequences are shown in Supplementary Figure 1. All primers used in this study were synthesized by Beijing Tsingke Biotechnology Co., Ltd (Nanning, China). Each 20μl reaction volume contained 2μl DNA template, 1μl of each primer (10μm), 10μl Master Mix, and 6μl ddH_2_O. The PCR protocol was as follows: 95°C for 5min; 30–35cycles of 95°C for 30s, 50–58°C for 30s, and 72°C for 30s; an extension step at 72°C for 5min; and holding at 4°C. QuickCutTM Hinf I (Takara) was used to digest the amplified PCR products at 37°C for 5min according to the manufacturer’s instructions. The PCR amplification and digested products were separated by 8% native polyacrylamide gel, voltage 300 v, and were run for 2h. Linkage was validated for 40 wax gourd germplasms comprising 25 and 15 wax gourd lines with green and white rind colors, respectively (Supplementary Figure 3).

## Results

### Inheritance and Phenotypic Characterization of Wax Gourd Skin Color

For the KX-2×YO-16 cross, the paternal KX-2 field phenotype was ink green, maternal YO-16 field phenotype was white, F_1_ phenotype was green, and F_2_ population underwent trait segregation (Table 1). The result was 180 green-skinned plants, 64 white-skinned plants, and a 3:1 green to white peel segregation ratio (*χ*^2^=0.197, *p*=0.657) among the 244 plants harvested. Of these, 118 were green-skinned and 115 were white-skinned in the BC_1_ population. This distribution conformed to a 1:1 segregation ratio (*χ*^2^=0.039, *p*=0.844).

**Table 1:** Segregation of peel color in wax gourd segregation populations.

Population	No. plants tested	Green:white	Expected Mendelian distribution	*χ* ^2^	*P*
KX-2×YO-16					
F_2_	244	180:64	3:1	0.197	0.657
BC_1_	233	118:115	1:1	0.039	0.844
GX-71×MY-1					
F_2_	1,244	930:314	3:1	0.039	0.844
BC_1_	472	238:234	1:1	0.034	0.854

*χ^2^ (0.05,1)=3.841; χ^2^=* $\overset{k}{\underset{i=1}{\sum }}\frac{{\left(O_{i}-E_{i}\right)}^{2}}{E_{i}}$*; k: Number of distinguishable categories, O_i_, Observations in Category i; E_i_, Expected value of category i*.

GX-71×MY-1 had a green paternal GX-71 field phenotype and white maternal MY-1 field phenotype. All F_1_ individuals were green, and trait segregation occurred in the F_2_ population. Of the 1,244 plants, 930 were green-skinned and 314 were white-skinned (Table 1). This distribution conformed to a 3:1 segregation ratio (*χ*^2^=0.039, p=0.844) and indicated Mendelian inheritance. In the BC_1_ population, 238 plants were green-skinned, and 234 plants were white-skinned. This distribution conformed to a 1:1 segregation ratio (*χ*^2^=0.034, *p*=0.854).

### Chlorophyll Content Determination and Chloroplast Observation

At 15days after four parentals pollination, wax gourd pericarps were subjected to microsection and observed under a light microscope. There was no significant difference between the parental lines in terms of the mature cell volume. However, there were only a few chloroplasts in the white skins and the peel appeared yellow (Figure 1B). In contrast, there were abundant chloroplasts in the green skins and the peel appeared green (Figure 1B). The microscopic and naked-eye observations were consistent.

Ethanol [95% (v/v)] was used to extract the chlorophyll and carotenoids from the four parentals and their F_1_ pericarps (15days after pollination), after which the pigment content was measured (Figure 1C). In KX-2× YO-16 hybridization, the chlorophyll a content of KX-2 was 11-fold higher than that of YO-16 and>2-fold higher than that of F_1_. The chlorophyll b content of KX-2 was eight-fold higher than that of YO-16 and 2.5-fold higher than that of F_1_. The total chlorophyll content of KX-2 was 9.7-fold higher than that of YO-16 and 2.4-fold higher than that of F_1_. The carotenoid content was significantly lower than the chlorophyll content in either green-skinned or white-skinned wax gourd. The carotenoid content of KX-2 was 28-fold higher than that of YO-16 and 2.7-fold higher than that of F_1_. In GX-71× MY-1 hybridization, the chlorophyll a content of GX-71 was 6.35-fold higher than that of MY-1 and 1.98-fold higher than that of their F_1._ The chlorophyll b content of GX-71 was 5.26-fold higher than that of MY-1 and 1.97-fold higher than that of F_1_. The total chlorophyll content of GX-71 was 5.96-fold higher than that of MY-1 and 1.98-fold higher than that of F_1_. The carotenoid content of GX-71 was 14.4-fold higher than that of MY-1 and 2.79-fold higher than that of F_1_.

### Preliminary Gene Mapping Results

**Figure 2:**
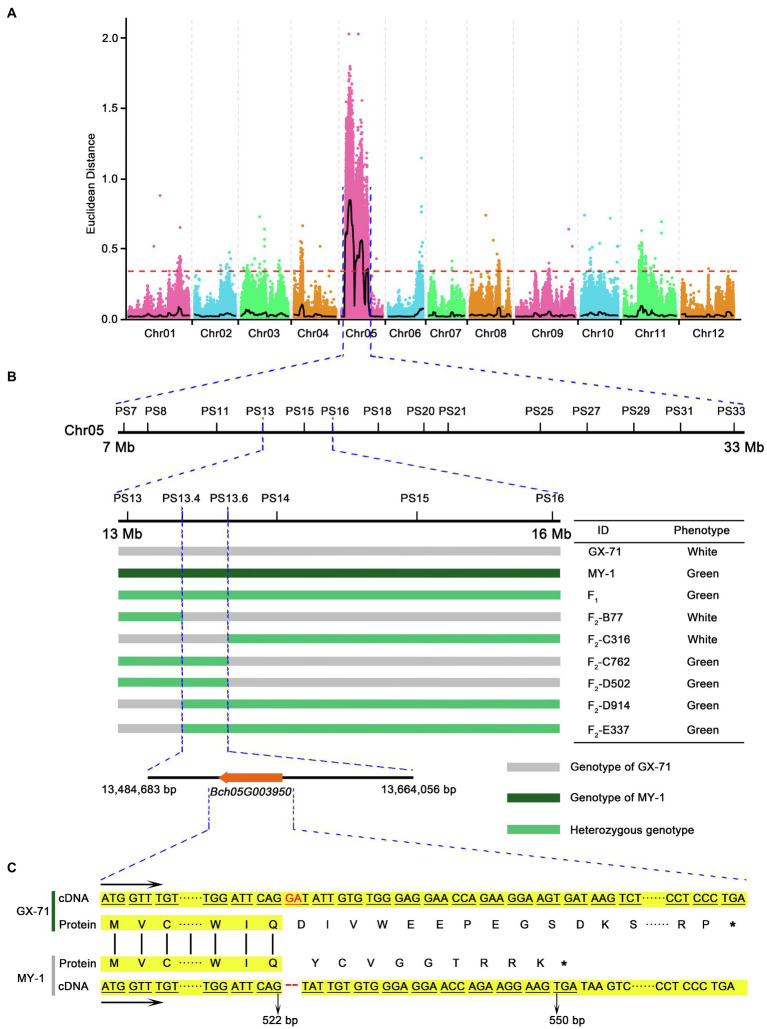
Mapping and cloning of *BhAPRR2* in wax gourd. **(A)** Manhattan plot for mapping of wax gourd peel color across two populations, GX-71 and MY-1. **(B)** Fine mapping of the candidate gene. The candidate gene was localized to a 179 Kb region between franking markers PS13.4 and PS13.6. **(C)** Comparison of coding sequence and protein sequence around the InDel site of parental lines (GX-71 and MY-1). The stop codon downstream of the 2bp InDel is shown.

In the present experiment, 60 F_2_ plants with extreme traits generated from GX-71 and MY-1 were resequenced to a 5× depth. Reads from each of the 30 extreme phenotype singletons were selected by phenotype, pooled to constitute gene mix pools, and used in BSA-seq. The output data were aligned with the wax gourd reference genome (GX-19) database and combined with parental resequencing data. Preliminary BSA mapping results for the white peel trait were obtained with the ED and SNP index association algorithms for BSA. There was a candidate region at the front end of Chr5 with a total length of 26Mb (Figure 2A). In parallel, 60 F_2_ plants with extreme traits were generated from an additional cross between KX-2 and YO-16 and used in resequencing to a 5× depth. The candidate region was also at the front end of Chr5 with a total length of 12.6Mb (Supplementary Figure 4). The interval was contained within 26Mb of the BSA preliminary mapping interval for F_2_ plants generated by GX-71 and MY-1. In order not to miss any possible genes, we chose the GX-71×MY-1 hybridization with a larger mapping interval for subsequent fine mapping. After the fine mapping, the F_2_ population of KX-2×YO-16 was used to verify the fine mapping results, so as to provide further evidence for the fine mapping results.

### Genetic Mapping of *BhAPRR2* to a 179 Kb Region

The F_2_ population resulting from GX-71×MY-1 hybridization was selected for gene fine mapping. In the mapped interval, 14 pairs of KASP markers (Supplementary Figure 2) were initially designed at 1–4Mb intervals according to the BSA results. A genotype-phenotype joint analysis was performed on the parents and 1,244F_2_ individuals. The interval was initially mapped to a region of 3.6Mb between PS13 and PS16. To identify and localize the genes controlling white wax gourd skin color, we expanded F_2_ plants to 5,000 plants. One PS11 marker and one PS18 marker were selected to flank PS13–PS16. Genotype-phenotype joint analysis was performed using these four markers on the 5,000F_2_ and 167 foregoing recombinant singletons and screened to yield 424 exchanged singletons. Further development of 3 pairs of KASP markers between PS13 and PS16, detection of the genotypes of the 424 recombinant singletons, and phenotypic data narrowed the candidate interval to PS13.4–PS13.6. The physical locations of these markers on Chr5 were 13,484,683 and 13,664,056, respectively, with a 179 Kb region between them (Figure 2B). Alignment with the wax gourd reference genome (GX-19) revealed the presence of a single candidate gene *Bch05G003950*, within this region. The Cucurbitaceae genome database combined with annotation information for the wax gourd reference genome (GX-19) identified this gene as *APRR2* encoding a two-component, response regulator-like protein previously reported for cucumber (Liu et al., 2016), melon, and watermelon (Oren et al., 2019). *Bch05G003950* was renamed as *BhAPRR2*.

### Gene Sequence Analysis

To evaluate the function of *BhAPRR2*, we downloaded its 1,556bp CDS from the wax gourd reference genome (GX-19), designed a primer pair (Supplementary Figure 1) to amplify the full-length CDS of all four parents by Primer 5 software, and performed gene cloning. The sequencing results were aligned using DNAMAN v.9 (Supplementary Figure 5). The green-skinned GX-71 material sequence agreed with the reference gene (GX-19). The green-skinned KX-2 material contained five SNP variations and three insertions relative to the reference gene. The sequences of the white-skinned accessions were concordant. Nevertheless, there was a two-base deletion at exon 7 of *BhAPRR2*, five SNP variations, and three insertions relative to the reference genome. We analyzed the putative amino acid sequence and found that the SNP variations did not form premature stop codons, the three base insertions did not cause a frameshift, and an extra Ser was added (Supplementary Figure 6). However, both white peel parents had a frameshift mutation comprising two bases (GA) deleted at exon 7. This mutation prematurely terminated translation to result in 335 fewer amino acids compared to the protein encoded by the reference genome (Figure 2C). Prediction of the *BhAPRR2* structure revealed that the missing 335 amino acids were localized to domains vital to protein function.

### Expression Analysis

**Figure 3:**
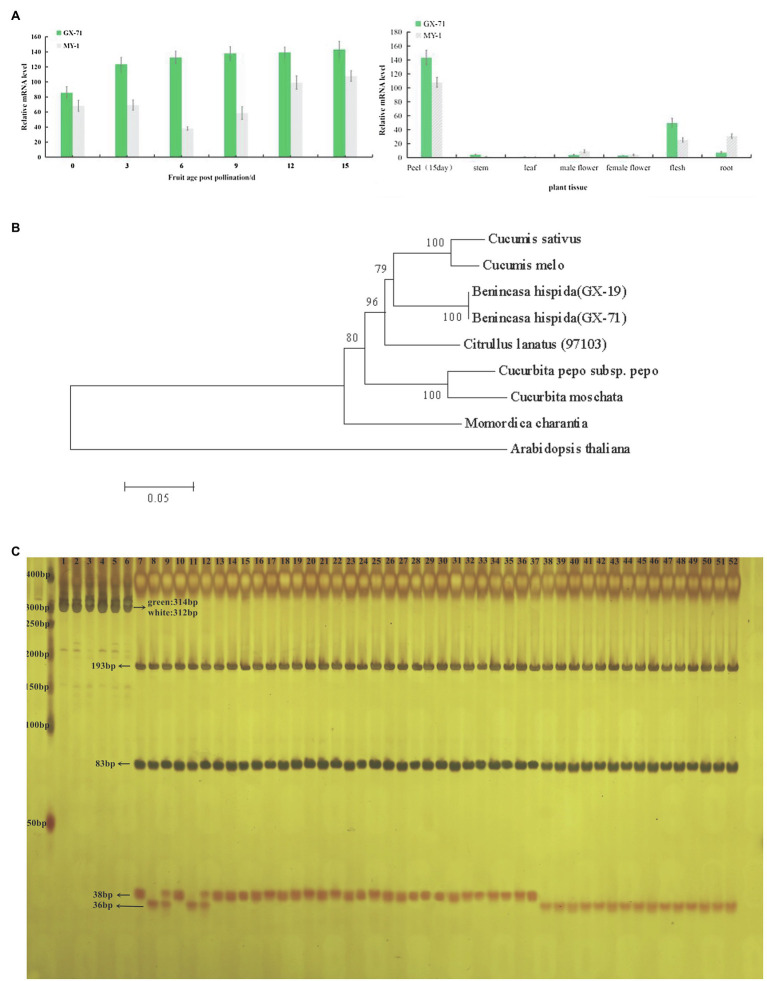
*BhAPRR2* expression and phylogenetic tree analysis and MAS detection.**(A)** Expression analysis of GX-71 and MY-1 in different periods in different tissues. ^*^, 0.01<*p*<0.05; ^**^, *p*<0.01. **(B)** Phylogenetic relationship of *APRR2* among selected species. **(C)** 40 inbred line germplasms tested the validity of a CAPS marker. 1–3: undigested PCR products of GX-71, MY-1, F_1_ of GX-71×MY-1, in turn; 4–6: undigested PCR products of KX-2,YO-16,F_1_ of KX-2×YO-16, in turn; 7–9: digested PCR products of GX-71, MY-1, F_1_ of GX-71×MY-1,in turn; 10–12: digested PCR products of KX-2,YO-16,F_1_ of KX-2×YO-16, in turn; 13–37: digested PCR products of 25 green-skinned inbred line germplasms; 38–52: digested PCR products of 15 white-skinned inbred line germplasms.

To reveal the expression profiles of *BhAPRR2* in wax gourd fruits at different developmental stages, RNA was extracted from the root, stem, leaf, male flower, female flower, and flesh at days 0, 3, 6, 9, 12, and 15 after pollination of GX-71 and MY-1 (Figure 3A). After reverse transcription, RT-qPCR was performed to determine the specific *BhAPRR2* expression profiles in the various wax gourd developmental periods and tissues. AP-2 complex subunit mu (*CAC, Bch05G003650*) was the internal reference gene. The results showed that GX-71 and MY-1 are significantly different in different developmental peels or in different tissues. Green-skinned wax gourd showed a high expression level from 0days after pollination, which gradually increased over time within 15days after pollination. The expression level of white-skinned wax gourd showed a downward trend from 0–6 days of pollination and gradually increased at 6days after pollination. Regardless of the period of fruit development, the green-skinned parent highly significantly differed from the white-skinned parent. At 0, 3, 6, 9, 12, and 15days after pollination, we observed 1.25-, 1.78-, 3.47-, 2.35-, 1.40-, and 1.33-fold differences, respectively. Meanwhile, the expression levels of KX-2 and YO-16 have the same trend. As the peel color differed more between KX-2 and YO-16, the amount of expression differed significantly (Supplementary Figure 7).

### Phylogenetic Analysis

We performed phylogenetic analysis using BLAST searches in the NCBI database and MEGA6 to clarify the relationship between *BhAPRR2* proteins and their homologs. The neighbor-joining tree revealed that wax gourd *BhAPRR2* had a close phylogenetic relationship with other cucurbits including cucumber, melon, watermelon, and pumpkin, which were located on the same branch (Figure 3B).

### Caps for Marker-Assisted Selection

We designed a codominant CAPS marker according to the *BhAPRR2* sequence in the wax gourd by Primer 5 software (Supplementary Figure 1). We validated this marker with 40 wax gourd accessions including 15 white-skinned and 25 green-skinned varieties (Supplementary Figure 3). The bands of 25 parts of green-skinned wax gourds were consistent with KX-2 and GX-71, whereas those of 15 parts of white-skinned wax gourds were consistent with YO-16 and MY-1. F_1_ has both male and female bands (Figure 3C). The corresponding material from each lane that PCR products were digested as shown in Supplementary Figure 2C.

## Discussion

Peel color is an important quality trait of wax gourd and indirectly influences consumer choice. In the present study, we used the wax gourd crosses KX-2×YO-16 and GX-71×MY-1 to identify and localize the genes regulating wax gourd peel color. The F_2_ from both crosses and the BC_1_ population exhibited green skin to white skin trait with segregation ratios of 3:1 and 1:1, respectively. Therefore, the genes regulating changes in fruit skin color have nuclear inheritance and green dominance over white. These findings are consistent with those of genetic pattern studies of cucurbit peel color reported by Li et al. (2019) and Liu (2018). Nevertheless, the phenotypically green peel color of the F_2_ population was inconsistent. The same trend was observed for white-skinned wax gourd. Although *BhAPRR2* has a major influence on wax gourd pericarp color, other genes may also have minor effects on this trait. Pericarp color is determined mainly by the anthocyanin and chlorophyll content and composition (Kayesh et al., 2013; Rosianskey et al., 2016). Chloroplast microscopy, and chlorophyll and carotenoid content measurements of four parentals and their F_1_ revealed that white-skinned wax gourd had fewer chloroplasts and lower chlorophyll a, chlorophyll b, total chlorophyll, and carotenoid content than green-skinned wax gourd. These results are consistent with the color changes observed by the naked eye and indicated that comparatively more chlorophyll accumulated in the green-skinned wax gourd. These results are also consistent with those reported by Wang et al. (2020) for green-skinned and white-skinned cucumber. Here, however, the difference between green- and white-skinned wax gourd cell size was not obvious at the same fold level. Wang et al. (2020) found that green-skinned materials have smaller epidermal cells than white-skinned materials. This finding is inconsistent with the results of the present study, possibly because of the relative differences in experimental material between these studies.

Here, two F_2_ populations were constructed from KX-2×YO-16 and GX-71×MY-1 and used to map candidate genes by BSA-seq and pooled sequencing. BSA-seq revealed peaks beyond the 95% confidence interval at the leading edge of Chr5. There was a 26Mb mapping interval for F_2_ plants from GX-71×MY-1. The candidate region obtained from KX-2 and YO-16 was included in the 26-Mb mapping interval. According to the BSA-seq results, we developed 14 pairs of KASP markers to screen 1,244F_2_ singletons and mapped the interval between PS13 and PS16. To narrow the candidate interval, we generated an F_2_ population containing 5,000 plants derived from GX-71 and MY-1. We flanked PS13 and PS16 with PS11 and PS18, genotyped 5,000F_2_ plants, and screened and identified 424 exchange plants. We developed three pairs KASP markers between PS13 and PS16 for the genotype-phenotype joint analysis of the 424 individuals. The candidate interval was compressed to 179 Kb between PS13.4 and PS13.6. Only *APRR2* (*Bch05G003950*) encoding a two-component, RR-like protein was present within this region. An earlier study identified a cluster of genes closely resembling ARR in *Arabidopsis thaliana* (Pan et al., 2013). Unlike true ARRs, however, they lack the invariant phosphor-acceptor aspartate site; hence, they are known as APRRs. *APRR2* is a member of the APRR family. Mapping analysis indicated that a nearly complete *APRR2* is required for APRR2 protein function in plants. Pan et al. (2013) demonstrated that *APRR1* is associated with plastid synthesis and pigment accumulation. The observed differences between the green- and white-skinned materials in terms of their chloroplast numbers and pigment content supported this conclusion. We sequenced *BhAPRR2* from all four parents and found that the white-skinned material had a deletion of two critical bases (GA; frameshift mutation) compared with the green-skinned material. Liu et al. (2016) and Oren et al. (2019) found that premature stop codon generation in the *APRR2* in dark material alters the peel color from dark to light. Liu (2018) also confirmed that *APRR2* regulates green peel formation. The *BhAPRR2* expression analysis performed on green-skinned wax gourd and white-skinned wax gourd indicated that in green-skinned peels, *BhAPRR2* was upregulated over time (0–15days) and was significantly higher than that in white-skinned wax gourd. Analysis of cucumber showed that the expression of *APRR2* was gradually upregulated over time after pollination until 12days and then was gradually downregulated (Liu et al., 2016). This finding is inconsistent with those of the present study. Cucumber peel changes from green to yellow during development. In contrast, wax gourd peel does not undergo this transformation. This explains the observed difference in cucumber and wax gourd regarding relative *APRR2* expression level. A CAPS marker was used to detect 40 inbred lines. The results of all inbred lines corresponded to their phenotypes, demonstrating that *BhAPRR2* is a powerful candidate gene for regulating peel color. Therefore, this marker can contribute to the application of marker-assisted selection.

The mechanism of *APRR2* is unclear. We predict that the two-base (GA) deletion in green-skinned melon caused a frame shift mutation, loss of 335 residues, and protein dysregulation. This mutation results in poor chloroplast development and chloroplast biosynthesis in green-skinned wax gourds and the resultant formation of white-skinned wax gourds. Nevertheless, the precise molecular mechanism requires further analysis. This study provides a theoretical basis for further evaluation of the regulatory mechanisms of the color genes in the fruit peel of wax gourd.

## Data Availability Statement

The data sets presented in this study can be found in online repositories. The names of the repository/repositories and accession number(s) can be found in the article/Supplementary Material.

## Author Contributions

LM: validation, formal analysis, investigation, and writing – original draft. ZL: resources and writing – review and editing. PW: conceptualization, methodology, and software. WY: supervision and project administration. ZC: investigation and software. JG: investigation and data curation. JC: investigation and data curation. All authors contributed to the article and approved the submitted version.

## Funding

This research was supported by the National Nature Science Foundation of China (31960593).

## Conflict of Interest

The authors declare that the research was conducted in the absence of any commercial or financial relationships that could be construed as a potential conflict of interest.

## Publisher’s Note

All claims expressed in this article are solely those of the authors and do not necessarily represent those of their affiliated organizations, or those of the publisher, the editors and the reviewers. Any product that may be evaluated in this article, or claim that may be made by its manufacturer, is not guaranteed or endorsed by the publisher.

## Footnotes

^1^ https://cucurbitgenomics.org

^2^ https://www.ncbi.nlm.nih.gov/
