# Supplementary material for: Identification and Application of BhAPRR2 Controlling Peel Colour in Wax Gourd (Benincasa hispida)
Source: Front Plant Sci. 2021 Oct 1;12:716772. doi: 10.3389/fpls.2021.716772 (PMC8517133; doi:10.3389/fpls.2021.716772)
Supplement: Supplementary file 1 [file Data_Sheet_1.PDF]

Supplementary Material

1 Supplementary Figures

Primer ID	Forward sequence (5′-3′)	Reverse sequence (5′-3′)	Application
CAC	aagtttgtggttgaggctgttg	gagcgaactccttcttgagtga	RT-qPCR for *CAC*
CQ	gtcgctctgctaccgagataag	cttgcacacacctccagaattg	RT-qPCR for *BhAPRR2*
PS CAPS	tcacttccttctggttcctcccacacaata	tgcagttgagttcttgctgaaaccactctc	MAS test
DAPRR2	atggtttgtactgccgacgatttac	tcagggaggtctggagccgttgatt	CDS of *BhAPRR2*

Supplementary Figure 1. Primers used in this study.

Primer ID	Primer X	Primer Y	Primer C	Application
PS7	gaaggtgaccaagttcatgctgtctgcattgtggcttcccatc	gaaggtcggagtcaacggattgtctgcattgtggcttcccatt	catccatggcatcctccaggaac	Reduce interval to 3.6 Mb
PS8	gaaggtgaccaagttcatgctggaactcccgacagttctttaca	gaaggtcggagtcaacggattgaactcccgacagttctttacg	aagaaccgtcgggagttcccag	Reduce interval to 3.6 Mb
PS11	gaaggtgaccaagttcatgctgttctcgctcttgtttggatcaaac	gaaggtcggagtcaacggattgttctcgctcttgtttggatcaaaa	aaattaccaaaacacatgagtgttacatagc	Reduce interval to 3.6 Mb and 179 Kb
PS13	gaaggtgaccaagttcatgcttaggtaaaaatgtcaacgttacagatc	gaaggtcggagtcaacggattttataggtaaaaatgtcaacgttacagatt	tcaacatgtcaagcttgcttgtggc	Reduce interval to 3.6 Mb and 179 Kb
PS13.4	gaaggtgaccaagttcatgctcttcatcttgatatcctccattctag	gaaggtcggagtcaacggatttcttcatcttgatatcctccattctaa	cggctggaatctacaatctacaatatattg	Reduce interval to 179 Kb
PS13.6	gaaggtgaccaagttcatgcttttggtggcaattagatgtttgttatg	gaaggtcggagtcaacggattgtttggtggcaattagatgtttgttatt	gtaaaaccattaatgtcatagataaacaatttcaca	Reduce interval to 179 Kb
PS14	gaaggtgaccaagttcatgctcaatcccacgattggtaggtttaca	gaaggtcggagtcaacggatttcccacgattggtaggtttacg	cggttaacgaattgaatcaaagaaacaaatc	Reduce interval to 179 Kb
PS15	gaaggtgaccaagttcatgctgaagcttggtcagccatggg	gaaggtcggagtcaacggattggaagcttggtcagccatgga	ccagcgaatttccagttctttcaacc	Reduce interval to 3.6 Mb
PS16	gaaggtgaccaagttcatgctcattagataacaccaaggatgactg	gaaggtcggagtcaacggattgtcattagataacaccaaggatgacta	gtgcagtcttgatcatggacttcag	Reduce interval to 3.6 Mb and 179 Kb
PS18	gaaggtgaccaagttcatgctgctaccaaattaatcaatcacacaatga	gaaggtcggagtcaacggattctaccaaattaatcaatcacacaatgg	catatagaaataagaatatatgcattaagctttagg	Reduce interval to 3.6 Mb and 179 Kb
PS20	gaaggtgaccaagttcatgctgaagactcttaaagactgaagtccg	gaaggtcggagtcaacggattgaagactcttaaagactgaagtcct	gaagttgaagtcttggaagaatacttgtatc	Reduce interval to 3.6 Mb

PS21	gaaggtgaccaagttcatgctccacc acaaagggctccaacat	gaaggtcggagtcaacggattcac cacaaagggctccaacac	gtggattcacttcgag gtctgcc	Reduce interval to 3.6 Mb
PS25	gaaggtgaccaagttcatgctgtgttg tggtttgttttacacttttgg	gaaggtcggagtcaacggattgtg ttgtggtttgttttacacttttga	atggaaggcaggga ggtgttgg	Reduce interval to 3.6 Mb
PS7	gaaggtgaccaagttcatgctaacaa ttcaaattaatccaaaaactgattctt	gaaggtcggagtcaacggattcaa ttcaaattaatccaaaaactgattct c	cataaggtccccttagt aactcaaaagg	Reduce interval to 3.6 Mb
PS29	gaaggtgaccaagttcatgctggcag catttggtacacataatcc	gaaggtcggagtcaacggattgg cagcatttggtacacataatcg	gaatctgtataaaaga ggggtagaccag	Reduce interval to 3.6 Mb
PS31	gaaggtgaccaagttcatgctccctat gatccccaagtatcca	gaaggtcggagtcaacggattccc tatgatccccaagtatccg	tggagcacttcgctttg taaagagg	Reduce interval to 3.6 Mb
PS33	gaaggtgaccaagttcatgctgaatta gtgaattgagtgaatttgaaatgg	gaaggtcggagtcaacggattgaa ttagtgaattgagtgaatttgaaatg a	gaaaaccccaaattta aacatttcatattgaaat tc	Reduce interval to 3.6 Mb

Supplementary Figure 2. KASP markers used in this study.

Number	Inbred line	Phenotype	Genotype *	Number	Inbred line	Phenotype	Genotype
7	GX-71	green	A	30	KF-4-3	green	A
8	MY-1	white	B	31	Hechijiegua	green	A
9	F_1 of GX-71×MY-1	green	H	32	7-2-1-4-2-2	green	A
10	KX-2	green	A	33	YO-13-2-2-1	green	A
11	YO-16	white	B	34	Mengshanjiegua	green	A
12	F_1 of KX-2×YO-16	green	H	35	Baidonggua	green	A
13	GK-3-4-3-2-1-3	green	A	36	YO-11-3-1	green	A
14	HX-1	green	A	37	GM-7-2-2-2	green	A
15	Rongshuijiegua	green	A	38	YMY-1-6-8-1	white	B
16	TL-1-2-2	green	A	39	YS-1-3-1-1	white	B
17	LF-1	green	A	40	YMY-1-5-0-1	white	B
18	Kx-2-3	green	A	41	YSB-1-3-1	white	B
19	HT-7-1-2-1	green	A	42	YO-16-1-2-6-1	white	B
20	GH-1-1-3	green	A	43	YMY-24-1-6-1	white	B

21	YO-2-2-1-6-1	green	A	44	YMY-2-2-1	white	B
22	GK-3-4-3	green	A	45	YSB-1-1-2	white	B
23	GF-7-1-1	green	A	46	JINGYUAN2HAO	white	B
24	YO-6-3-1-2-1	green	A	47	YMY-2-2	white	B
25	7-2-1-2	green	A	48	YO-11-1-1	white	B
26	GD-1	green	A	49	YO-24-1-3-1	white	B
27	NH-2	green	A	50	YO-2-2-3	white	B
28	GF-71	green	A	51	YS-4-5-1	white	B
29	M-1-2-1	green	A	52	YL-1-1-1	white	B

Supplementary Figure 3. MAS testing in 40 *Benincasa hispida* inbred lines and four parent lines and F_1using PS CAPS.*A representatives genotype of male parent(green),B representatives genotype of female parent(white).H representatives Heterozygote.

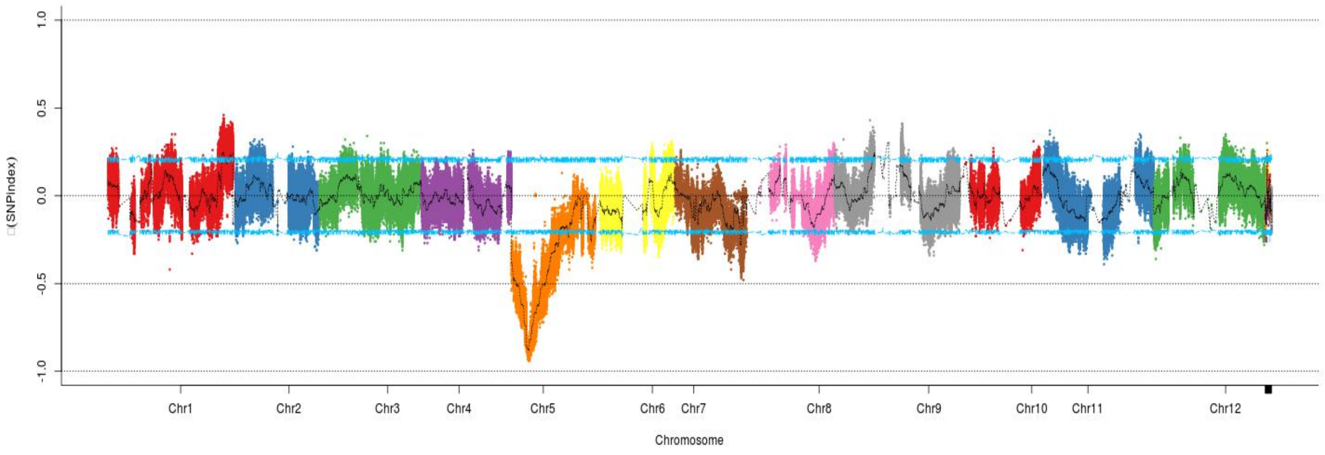

Supplementary Figure 4. Manhattan plot for mapping of wax gourd peel colour across two populations, KX-2 and YO-16.

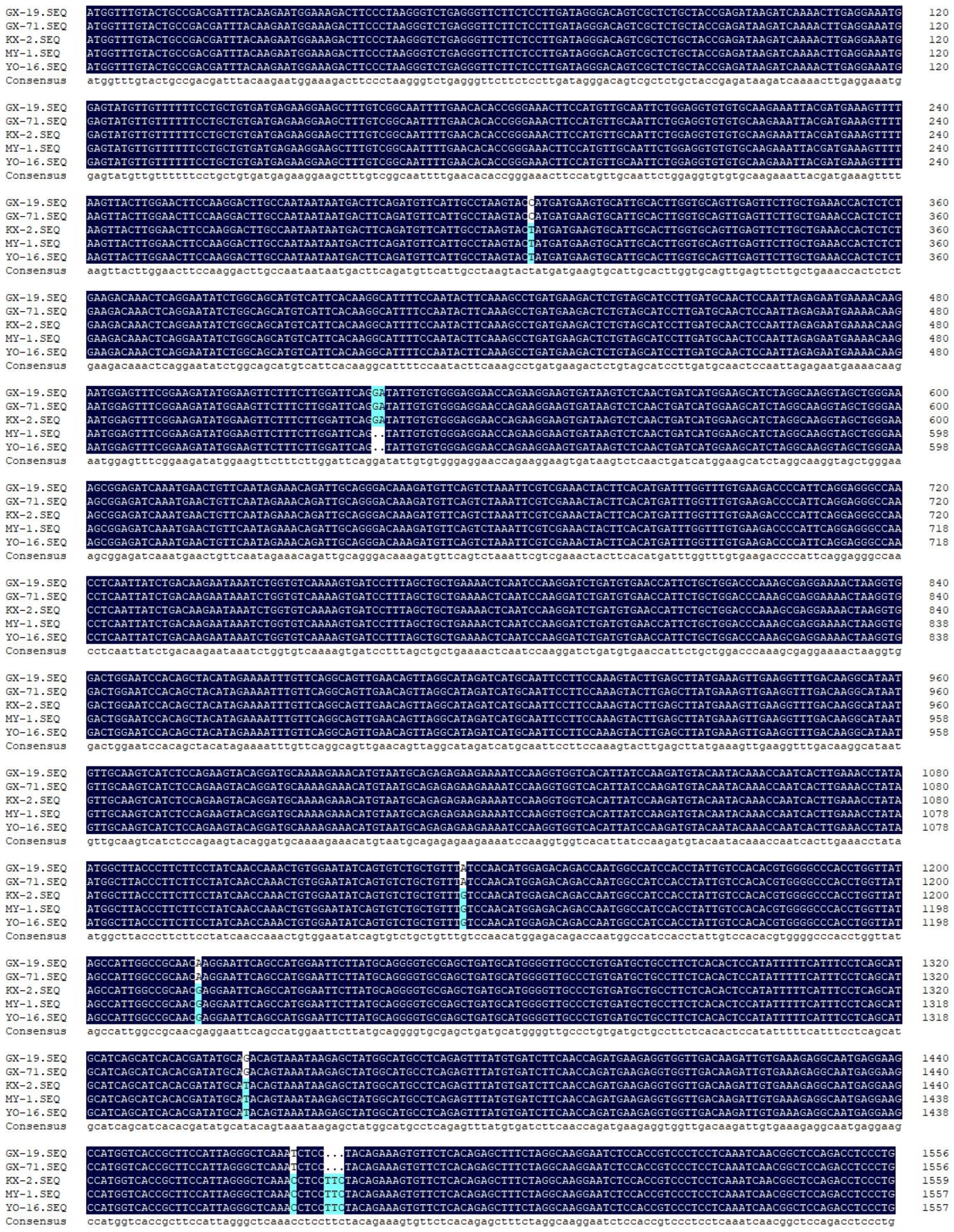

Supplementary Figure 5. Full-length coding sequence of four parents.

```
GX-19.seq_Translation   MVCTADDLQEWKDFPKGLRVLLLDRDSRSATEIRSKLEEMEYVVFSCCDEKEALSAILNTPGNFHVAILEVCARNYDESFKLLGTSKDLPIIMTSDVHCLSTMMKCIALGAVEFLLKPLS   120
GX-71.seq_Translation   MVCTADDLQEWKDFPKGLRVLLLDRDSRSATEIRSKLEEMEYVVFSCCDEKEALSAILNTPGNFHVAILEVCARNYDESFKLLGTSKDLPIIMTSDVHCLSTMMKCIALGAVEFLLKPLS   120
KX-2.seq_Translation    MVCTADDLQEWKDFPKGLRVLLLDRDSRSATEIRSKLEEMEYVVFSCCDEKEALSAILNTPGNFHVAILEVCARNYDESFKLLGTSKDLPIIMTSDVHCLSTMMKCIALGAVEFLLKPLS   120
MY-1.seq_Translation    MVCTADDLQEWKDFPKGLRVLLLDRDSRSATEIRSKLEEMEYVVFSCCDEKEALSAILNTPGNFHVAILEVCARNYDESFKLLGTSKDLPIIMTSDVHCLSTMMKCIALGAVEFLLKPLS   120
YO-16.seq_Translation   MVCTADDLQEWKDFPKGLRVLLLDRDSRSATEIRSKLEEMEYVVFSCCDEKEALSAILNTPGNFHVAILEVCARNYDESFKLLGTSKDLPIIMTSDVHCLSTMMKCIALGAVEFLLKPLS   120
Consensus               mvctaddlqewkdfpkglrvllldrdsrsateirskleemeyvvfsccdekealsailntpgnfhvailevcarnydesfkllgtskdlpiimtsdvhclstmmkcialgavefllkpls

GX-19.seq_Translation   EDKLRNIWQHVIHKAFSNTSKPDEDSVASLMQLQLENENKNGVSEDMEVLSWIQDIVWEEPEGSDKSQLIMEASRQGSWESGDQMNCSIETDCRDKDVQSKFVETTSHDLVCEDPIQEGQ   240
GX-71.seq_Translation   EDKLRNIWQHVIHKAFSNTSKPDEDSVASLMQLQLENENKNGVSEDMEVLSWIQDIVWEEPEGSDKSQLIMEASRQGSWESGDQMNCSIETDCRDKDVQSKFVETTSHDLVCEDPIQEGQ   240
KX-2.seq_Translation    EDKLRNIWQHVIHKAFSNTSKPDEDSVASLMQLQLENENKNGVSEDMEVLSWIQDIVWEEPEGSDKSQLIMEASRQGSWESGDQMNCSIETDCRDKDVQSKFVETTSHDLVCEDPIQEGQ   240
MY-1.seq_Translation    EDKLRNIWQHVIHKAFSNTSKPDEDSVASLMQLQLENENKNGVSEDMEVLSWIQYCVGGTRRK.........................................................   183
YO-16.seq_Translation   EDKLRNIWQHVIHKAFSNTSKPDEDSVASLMQLQLENENKNGVSEDMEVLSWIQYCVGGTRRK.........................................................   183
Consensus               edklrniwqhvihkafsntskpdedsvaslmqlqlenenkngvsedmevlswiqdivweepegsdksqlimeasrqgswesgdqmncsietdcrdkdvqskfvettshdlvcedpiqegq

GX-19.seq_Translation   PQLSDKNKSGVKSDPLAAENSIQGSDVNHSAGPKARKTKVDWNPQLHRKFVQAVEQLGIDHAIPSKVLELMKVEGLTRHNVASHLQKYRMQKKHVMQREENPRWSHYPRCTIQTNHLKPI   360
GX-71.seq_Translation   PQLSDKNKSGVKSDPLAAENSIQGSDVNHSAGPKARKTKVDWNPQLHRKFVQAVEQLGIDHAIPSKVLELMKVEGLTRHNVASHLQKYRMQKKHVMQREENPRWSHYPRCTIQTNHLKPI   360
KX-2.seq_Translation    PQLSDKNKSGVKSDPLAAENSIQGSDVNHSAGPKARKTKVDWNPQLHRKFVQAVEQLGIDHAIPSKVLELMKVEGLTRHNVASHLQKYRMQKKHVMQREENPRWSHYPRCTIQTNHLKPI   360
MY-1.seq_Translation    ........................................................................................................................   183
YO-16.seq_Translation   ........................................................................................................................   183
Consensus               pqlsdknksgvksdplaaensiqgsdvnhsagpkarktkvdwnpqlhrkfvqaveqlgidhaipskvlelmkvegltrhnvashlqkyrmqkkhvmqreenprwshyprctiqtnhlkpi

GX-19.seq_Translation   MAYPSSYQPNCGISVSAVYPTWRQTNGHPPIVHTWGPPGYS.HWPQQGIQPWNSYAGVRADAWGCPVMLPSHTPYFSFPQHASASHDMQTVNKSYGMPQSLCDLQPDEEVVDKIVKEAMR   479
GX-71.seq_Translation   MAYPSSYQPNCGISVSAVYPTWRQTNGHPPIVHTWGPPGYS.HWPQQGIQPWNSYAGVRADAWGCPVMLPSHTPYFSFPQHASASHDMQTVNKSYGMPQSLCDLQPDEEVVDKIVKEAMR   479
KX-2.seq_Translation    MAYPSSYQPNCGISVSAVCPTWRQTNGHPPIVHTWGPPGYS.HWPQRGIQPWNSYAGVRADAWGCPVMLPSHTPYFSFPQHASASHDMHTVNKSYGMPQSLCDLQPDEEVVDKIVKEAMR   479
MY-1.seq_Translation    ........................................................................................................................   183
YO-16.seq_Translation   ........................................................................................................................   183
Consensus               maypssyqpncgisvsav ptwrqtnghppivhtwgppgys hwpq giqpwnsyagvradawgcpvmlpshtpyfsfpqhasashdm tvnksygmpqslcdlqpdeevvdkivkeamr

GX-19.seq_Translation   KPWSPLPLGLKSP.TESVLTELSRQGISTVPPQINGSRP   517
GX-71.seq_Translation   KPWSPLPLGLKSP.TESVLTELSRQGISTVPPQINGSRP   517
KX-2.seq_Translation    KPWSPLPLGLKPPSTESVLTELSRQGISTVPPQINGSRP   518
MY-1.seq_Translation    .......................................   183
YO-16.seq_Translation   .......................................   183
Consensus               kpwsplplglk p tesvltelsrqgistvppqingsrp
```

Supplementary Figure 6. Protein sequences of four parents.

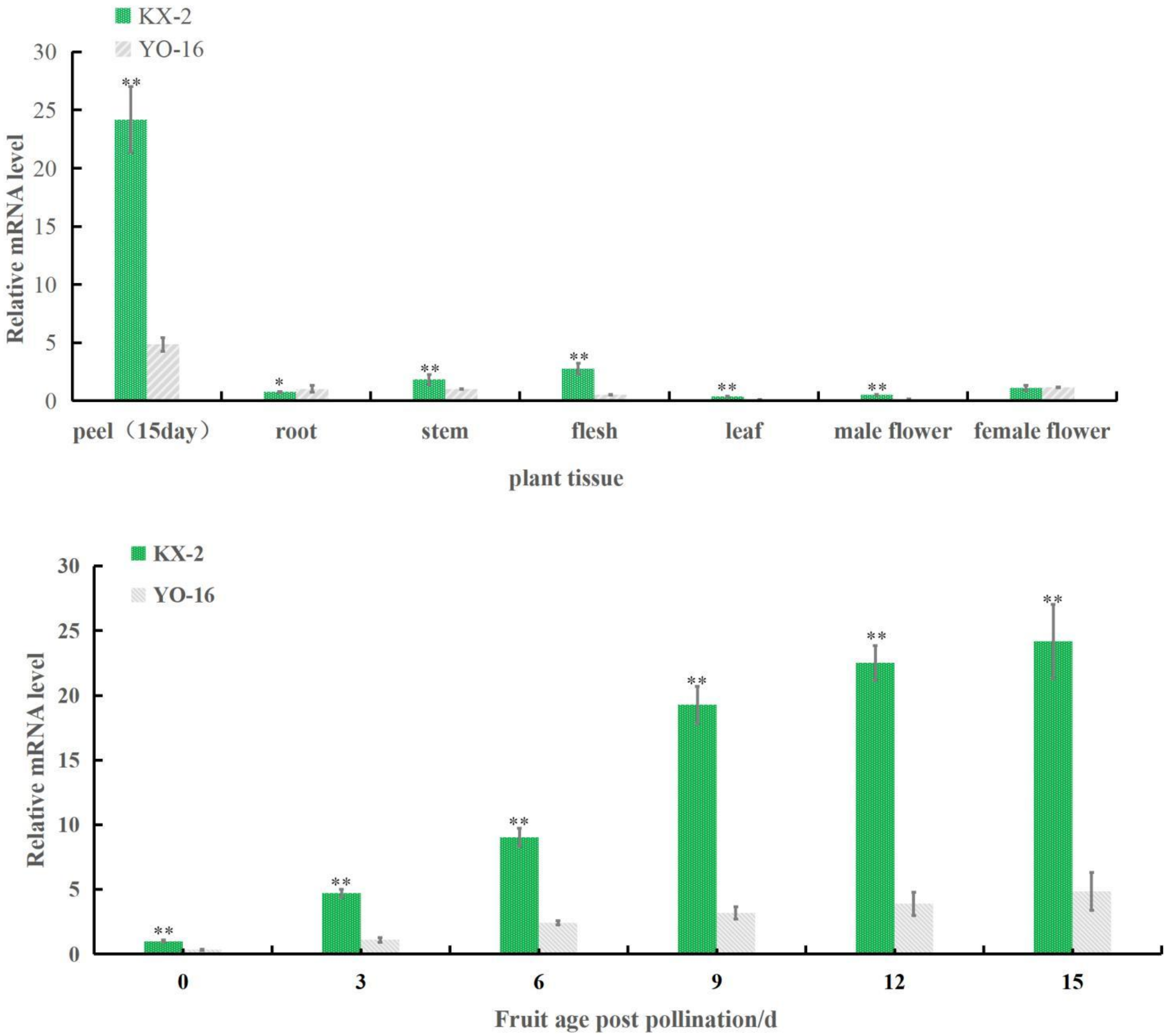

Supplementary Figure 7. Expression analysis of KX-2 and YO-16 in different periods in different tissues. *: 0.01 < P < 0.05, **: P < 0.01.

**Species**	**Accession**
Cucumis sativus	AMJ39435.1
Cucumis melo	XP_008465934.1
Momordica charantia	XP_022156603.1
Cucurbita pepo subsp. pepo	XP_023554702.1
Cucurbita moschat	XP_022952808.1
Cucurbita maxima	XP_022971967.1
Citrullus lanatus (Watermelon (97103) v2)	Cla97C09G175170
Benincasa hispida	XP_038877823.1
Arabidopsis thaliana	NP_567548

Supplementary Figure 8. Accession numbers of amino acid sequences downloaded from NCBI
